# 75 years of IUCr Journals: 1948 to 2023, an Editor-in-Chief’s perspective

**DOI:** 10.1107/S205225252300670X

**Published:** 2023-09-01

**Authors:** Andrew Allen

**Affiliations:** a Materials Measurement Science Division, National Institute of Standards and Technology, 100 Bureau Drive, Gaithersburg, MD 20899-8520, USA

**Keywords:** IUCr journals, *Acta Crystallographica*, *Journal of Applied Crystallography*, *Journal of Synchrotron Radiation*, *IUCrJ*, *IUCrData*, International Union of Crystallography

## Abstract

On the 75th anniversary of the International Union of Crystallography (IUCr) and its journals, a brief history is presented, highlighting selected publications, based on citations and on other criteria of note for each journal. Emerging from the pandemic, prospects for the future are considered, especially in the context of the ongoing transformation to open-access research publication affecting all scientific research journals.

## Abstract 2

The International Union of Crystallography (IUCr) and its journals are celebrating their 75th anniversary. In this editorial, some of the publication achievements are highlighted and prospects for the future reviewed in an emerging open-access world.

## 1. The International Union of Crystallography (IUCr) and its journals

The IUCr came into being 75 years ago in 1948 and others will recount the history of its origins as part of these 75th Anniversary celebrations. Also, please see earlier histories of the IUCr (Kamminga, 1989 ▸) and of the IUCr journals, especially on their 60th anniversary (Authier, 2009 ▸). Right from the start, the IUCr recognized the importance of high-quality publication of crystallographic research and structural data with its own journal, *Acta Crystallographica* (*Acta Cryst.*). *Acta Cryst.* remained the sole IUCr journal for some 20 years, but in 1968, it was expanded into two sections: *Acta Cryst. A* for crystal physics, diffraction, and theoretical and general crystallography, and *Acta Cryst. B* for structural crystallography and crystal chemistry. At around this same time (and following a decision made earlier at the 1963 IUCr General Assembly) a new journal was founded: *Journal of Applied Crystallography* for reporting methods, apparatus, problems and discoveries in applied crystallography. In 1983, *Acta Cryst.* was further expanded into three sections with the founding of *Acta Cryst. C* to handle crystal structure communications, and in 1993 it was expanded again with *Acta Cryst. D* to provide a needed home for the increasing number of biological crystallography submissions. The following year, in October 1994, another new IUCr journal was founded: the *Journal of Synchrotron Radiation* for submissions across the whole remit of synchrotron science (and later free-electron laser X-ray sources).

A key development was the introduction of online journal versions in 1999. Up to this point, both the review process and journal publication, itself, took place almost exclusively in hardcopy form, and this author can just remember (as a new Co-editor in 2002) inheriting a ‘Not for publication – for review only’ stamp to use on hardcopy submissions received and then sent out to review. Over the following five years or so, the review system became entirely electronic and eventually web-based. A number of other important changes came to the journals in this period, such as the advent of an open-access option for authors in 2004. Partly to take advantage of these changes, two new sections were added to *Acta Cryst.*: *Acta Cryst. E* in 2001 to carry structure reports online, and *Acta Cryst. F* in 2005 for rapid structural biology communications. In 2008, *Acta Cryst. E* became the first IUCr journal to flip to ‘open-access only’ while in 2014 all the IUCr journals went to online publication only. Also, coinciding with the United Nations declaration of the *International Year of Crystallography* in 2014, the IUCr launched a new fully open-access journal, *IUCrJ*, to attract high-quality cross-cutting papers of broad scientific significance from all areas of structural science and crystallography. *IUCrJ* now covers seven main subject areas with papers pre-selected by the Main Editors prior to review.

Completing the complement of IUCr journals is *IUCrData*, fully open access from its inception in 2016, at least initially to take data reports formerly submitted to *Acta Cryst. E*. This was associated with the transformation of *Acta Cryst. E* from a journal focused on Structure Reports Online to one more focused on Crystallographic Communications. The transformation of *Acta Cryst. E* was necessitated in part by its removal from the main journal citation index in 2012. Both journals have continued to develop and evolve over the years with a *Raw Data Letters* section recently started in *IUCrData*, while *Acta Cryst. E* has become fully re-indexed with an impact factor once more from 2023. Meanwhile in 2022, the *Journal of Synchrotron Radiation* flipped to open access only so that in 2023 there are four IUCr journals fully open access while six remain hybrid, publishing both open-access papers and papers accessed via traditional subscription arrangements.

In what follows, selected papers are highlighted, based on citations and other criteria of note to the Main and Managing Editors of the journals. Some more recent papers are included to encompass new achievements and point to potentially significant future developments.

## 2. Brief surveys of selected significant papers appearing in the IUCr journals over the years

### 2.1. IUCrJ

The scope of *IUCrJ* has increased to cover seven main subject areas: *Biology and Medicine*, *Chemistry and Crystal Engineering*, *CryoEM*, *Electron Crystallography*, *Materials and Computation*, *Neutron and Synchrotron Science and Technology*, and *Physics and Free Electron Science and Technology*. The titles of the highly cited articles listed below indicate the range of high-impact cross-cutting research that has been reported in *IUCrJ* since its inception.

A density functional theory (DFT) energy calibration tool, *CrystalExplorer*,^1^ extended to many molecular crystals: *Crystal­Explorer model energies and energy frameworks: extension to metal coordination compounds, organic salts, solvates and open-shell systems.* Mackenzie, C. F., Spackman, P. R., Jayatilaka, D. & Spackman, M. A. (2017). *IUCrJ*, **4**, 575–587. https://doi.org/10.1107/S205225251700848X.

An advanced server facility to substantially improve structure models prior to submission to the Protein Database (PDB): *The PDB_REDO server for macromolecular structure model optimization.* Joosten, R. P., Long, F., Murshudov, G. N. & Perrakis, A. (2014). *IUCrJ*, **1**, 213–220. https://doi.org/10.1107/S2052252514009324.

An enhanced model for small-angle X-ray scattering (SAXS) from biological macromolecules: *Advanced ensemble modelling of flexible macromolecules using X-ray solution scattering.* Tria, G., Mertens, H. D. T., Kachala, M. & Svergun, D. I. (2015). *IUCrJ*, **2**, 207–217. https://doi.org/10.1107/S205225251500202X.

A new method to estimate particle motion and address beam damage effects in cryo-EM: *A Bayesian approach to beam-induced motion correction in cryo-EM single-particle analysis.* Zivanov, J., Nakane, T. & Scheres, S. H. W. (2019). *IUCrJ*, **6**, 5–17. https://doi.org/10.1107/S205225251801463X.

Fingerprinting molecular interactions in crystalline environments to derive enrichment ratios: *The enrichment ratio of atomic contacts in crystals, an indicator derived from the Hirshfeld surface analysis.* Jelsch, C., Ejsmont, K. & Huder, L. (2014). *IUCrJ*, **1**, 119–128. https://doi.org/10.1107/S2052252514003327.

An early review of opportunities and challenges of serial femtosecond crystallography: *Serial femtosecond crystallography: the first five years.* Schlichting, I. (2015). *IUCrJ*, **2**, 246–255. https://doi.org/10.1107/S205225251402702X.

Enhanced developments of serial crystallography methods to upgraded facilities: *Room-temperature macromolecular serial crystallography using synchrotron radiation.* Stellato, F., Oberthur, D., Liang, M. N., Bean, R., Gati, C., Yefanov, O., Barty, A., Burkhardt, A., Fischer, P., Galli, L., Kirian, R. A., Meyer, J., Panneerselvam, S., Yoon, C. H., Chervinskii, F., Speller, E., White, T. A., Betzel, C. Meents, A. & Chapman, H. N. (2014). *IUCrJ*, **1**, 204–212. https://doi.org/10.1107/S2052252514010070.

Grazing-incidence small-angle and wide-angle scattering to probe soft-matter nanostructures: *Advanced grazing-incidence techniques for modern soft-matter materials analysis.* Hexemer, A. & Muller-Buschbaum, P. (2015). *IUCrJ*, **2**, 106–125. https://doi.org/10.1107/S2052252514024178.

Orienting organic molecules within porous crystal sponges to enable conventional X-ray diffraction: *The crystalline sponge method updated.* Hoshino, M., Khutia, A., Xing, H. Z., Inokuma, Y. & Fujita, M. (2016). *IUCrJ*, **3**, 139–151. https://doi.org/10.1107/S2052252515024379.

State-of-the-art X-ray free-electron lasers to outrun radiation damage effects in structural biology: *XFELs for structure and dynamics in biology.* Spence, J. C. H. (2017). *IUCrJ*, **4**, 322–339. https://doi.org/10.1107/S2052252517005760.

Deep machine learning: *Classification of crystal structure using a convolutional neural network.* Park, W. B., Chung, J., Jung, J., Sohn, K., Singh, S. P., Pyo, M., Shin, N. & Sohn, K. S. (2017). *IUCrJ*, **4**, 486–494. https://doi.org/10.1107/S205225251700714X.

Recent papers further demonstrate the impact of *IUCrJ* both in its most recently added subject area of *Electron Crystallography* and elsewhere in publishing structural science relevant to the fight against COVID-19:

A study revealing two possible crystallization pathways for an active pharmaceutical ingredient: *Revealing the early stages of carbamazepine crystallization by cryoTEM and 3D electron diffraction.* Broadhurst, E. T., Xu, H., Parsons, S. & Nudelman, F. (2021). *IUCrJ*, **8**, 860–866. https://doi.org/10.1107/S2052252521010101.

Over 100 models of major drug-design targets for COVID-19 assembled and validated: *Crystallographic models of SARS-CoV-2 3CLpro: in-depth assessment of structure quality and validation.* Jaskolski, M., Dauter, Z., Shabalin, I. G., Gilski, M., Brzezinski, D., Kowiel, M., Rupp, B. & Wlodawer, A. (2021). *IUCrJ*, **8**, 238–256. https://doi.org/10.1107/S2052252521001159.

### 2.2. Acta Cryst. A: Foundations and Advances

*Acta Cryst. A* has two sections: a rapid-publication *Advances* section for articles describing original research of high potential impact and the traditional *Foundations* section. Articles submitted as *Advances* are pre-screened for likely impact and interest by the Main Editors. The wide range of significant papers that have been published in *Acta Cryst. A* is represented by the following list of selected well cited papers with their main subject areas.

Structures and computation: *A short history of SHELX.* Sheldrick, G. M. (2008). *Acta Cryst. A* **64**, 112–122. https://doi.org/10.1107/S0108767307043930.

Mathematical crystallography and computation: *Bilbao Crystallographic Server. II. Representations of crystallographic point groups and space groups.* Aroyo, M. I., Kirov, A., Capillas, C., Perez-Mato, J. M. & Wondratschek, H. (2006). *Acta Cryst. A* **62**, 115–128. https://doi.org/10.1107/S0108767305040286.

Computational methods and materials: *Ab initio structure solution by charge flipping.* Oszlányi, G. & Sütő, A. (2004). *Acta Cryst.* A**60**, 134–141. https://doi.org/10.1107/S0108767303027569.

Powder diffraction: *Whole powder pattern modelling.* Scardi, P. & Leoni, M. (2002). *Acta Cryst.* A**58**, 190–200. https://doi.org/10.1107/S0108767301021298.

Local structure and nanostructure: *Complex modelling: a strategy and software program for combining multiple information sources to solve ill posed structure and nanostructure inverse problems.* Juhás, P., Farrow, C., Yang, X., Knox, K. & Billinge, S. (2015). *Acta Cryst.* A**71**, 562–568. https://doi.org/10.1107/S2053273315014473.

Electron diffraction: *Structure refinement using precession electron diffraction tomography and dynamical diffraction: theory and implementation.* Palatinus, L., Petříček, V. & Corrêa, C. A. (2015). *Acta Cryst.* A**71**, 235–244. https://doi.org/10.1107/S2053273315001266.

Quasicrystals: *The structure of a decagonal Al_72_Ni_20_Co_8_ quasicrystal.* Takakura, H., Yamamoto, A. & Tsai, A. P. (2001). *Acta Cryst.* A**57**, 576–585. https://doi.org/10.1107/S0108767301007942.

Quantum crystallography: *X-ray structure refinement using aspherical atomic density functions obtained from quantum-mechanical calculations.* Jayatilaka, D. & Dittrich, B. (2008). *Acta Cryst.* A**64**, 383–393. https://doi.org/10.1107/S0108767308005709.

Traditional crystallography: *Revised effective ionic radii and systematic studies of interatomic distances in halides and chalcogenides.* Shannon, R. D. (1976). *Acta Cryst.* A**32**, 751–767. https://doi.org/10.1107/S0567739476001551.

Traditional crystallography: *On enantiomorph-polarity estimation.* Flack, H. D. (1983). *Acta Cryst.* A**39**, 876–881. https://doi.org/10.1107/S0108767383001762.

*Acta Cryst A* continues to publish papers to address how recent advances are likely to change the practice of crystallography. Two recent examples focus on the need to deal with very large, rapidly accumulated, datasets and to develop appropriate machine-learning methods in structural science research:

*Crystal diffraction prediction and partiality estimation using Gaussian basis functions.* Brehm, W., White, T. & Chapman, H. N. (2023). *Acta Cryst. A* **79**, 145–162. https://doi.org/10.1107/S2053273323000682.

*Towards a machine-readable literature: finding relevant papers based on an uploaded powder diffraction pattern.* Özer, B., Karlsen, M. A., Thatcher, Z., Lan, L., McMahon, B., Strickland, P. R., Westrip, S. P., Sang, K. S., Billing, D. G., Ravnsbaek, D. B. & Billinge, S. J. L. (2022). *Acta Cryst. A* **78**, 386–394. https://doi.org/10.1107/S2053273322007483.

### 2.3. Acta Cryst. B: Structural Science, Crystal Engineering and Materials

*Acta Cryst. B* welcomes articles on structural science of compounds and materials in the widest sense. It also welcomes contributions focusing on all aspects of crystal growth related to structural science, crystal engineering and materials. Indeed, a new section focused explicitly on *Crystal Growth* was started in late 2021.

The most highly cited articles in *Acta Cryst. B* are associated with structural databases such as the Cambridge Structural Database (CSD) and the complementary Inorganic Crystal Structure Database (ICSD) – see papers listed below. Collectively, they describe a massive, curated resource for organic and metal–organic structures, underpinning a significant category of articles using the databases to investigate topics such as trends in chemical bonding, patterns of intermolecular contacts and packing motifs.

*The Cambridge Structural Database.* Groom, C. R., Bruno, I. J., Lightfoot, M. P. & Ward, S. C. (2016). *Acta Cryst.* B**72**, 171–179. https://doi.org/10.1107/S2052520616003954. This follows on from the earlier paper: *The Cambridge Structural Database: a quarter of a million crystal structures and rising.* Allen, F. H. (2002). *Acta Cryst.* B**58**, 380–388. https://doi.org/10.1107/S0108768102003890.

*New developments in the Inorganic Crystal Structure Database (ICSD): accessibility in support of materials research and design.* Belsky, A., Hellenbrandt, M., Karen, V. L. & Luksch, P. (2002). *Acta Cryst.* B**58**, 364–369. https://doi.org/10.1107/S0108768102006948.

Another important area for *Acta Cryst. B* has been in papers on absolute structure. Building on earlier work, largely between 1983 and 2008, the paper below was a significant and highly cited update that has led to enhanced tools for the determination of absolute structure.

*Use of intensity quotients and differences in absolute structure refinement.* Parsons, S., Flack, H. D. & Wagner, T. (2013). *Acta Cryst.* B**69**, 249–259. https://doi.org/10.1107/S2052519213010014.

Other well cited series of *Acta Cryst. B* papers have followed on from initial papers in key subject areas, and *Acta Cryst. B* remains one of the most diverse of the IUCr journals. Selected papers from one such series on band-valence parameters are listed below, the first being one of the earliest highly cited papers in the journal.

*Bond-valence parameters obtained from a systematic analysis of the Inorganic Crystal Structure Database.* Brown, I. D. & Altermatt, D. (1985). *Acta Cryst.* B**41**, 244–247. https://doi.org/10.1107/S0108768185002063.

*Bond-valence parameters for solids.* Brese, N. E. & O’Keeffe, M. (1991). *Acta Cryst.* B**47**, 192–197. https://doi.org/10.1107/S0108768190011041.

*Comprehensive derivation of bond-valence parameters for ion pairs involving oxygen.* Gagne, O. C. & Hawthorne, F. C. (2015). *Acta Cryst.* B**71**, 562–578. https://doi.org/10.1107/S2052520615016297.

In recent years, *Acta Cryst. B* has published major contributions to research on many modern materials of interest. It is not possible to capture the breadth of the research covered in a short list, but the titles of the following well cited papers speak for themselves.

*Model structures for C-(A)-S-H(I).* Richardson, I. G. (2014). *Acta Cryst.* B**70**, 902–923. https://doi.org/10.1107/S2052520614021982.

*Crystalline metal–organic frameworks (MOFs): synthesis, structure and function.* Dey, C., Kundu, T., Biswal, B. P., Mallick, A. & Banerjee, R. (2014). *Acta Cryst.* B**70**, 3–10. https://doi.org/10.1107/S2052520613029557.

*Validation of molecular crystal structures from powder diffraction data with dispersion-corrected density functional theory (DFT-D).* van de Streek, J. & Neumann, M. A. (2014). *Acta Cryst.* B**70**, 1020–1032. https://doi.org/10.1107/S2052520614022902.

*SoftBV – a software tool for screening the materials genome of inorganic fast ion conductors.* Chen, H. M., Wong, L. L. & Adams, S. (2019). *Acta Cryst.* B**75**, 18–33. https://doi.org/10.1107/S2052520618015718.

*X-ray constrained wavefunctions based on Hirshfeld atoms. I. Method and review.* Davidson, M. L. Grabowsky, S. & Jayatilaka, D. (2022). *Acta Cryst.* B**78**, 312–332. https://doi.org/10.1107/S2052520622004097.

### 2.4. Acta Cryst. C: Structural Chemistry

Over the past few years, *Acta Cryst C* has transitioned from simply handling crystal structure communications to publishing exciting science with structural content and important results relating to the chemical sciences. However, *Acta Cryst. C* has published impactful papers over its entire history. In this case, a chronological list of selected papers helps to illustrate how its scope has evolved over the years.

Highlighting neutron diffraction as a useful tool in solid-state materials chemistry: *Cobalt(III) lithium oxide, CoLiO_2_: structure refinement by powder neutron diffraction.* Orman, H. J. & Wiseman, P. J. (1984). *Acta Cryst.* C**40**, 12–14. https://doi.org/10.1107/S0108270184002833.

Crystallographic studies correlating phase transitions with changes in magnetic properties: *The structure of a new magnetic phase related to the sigma phase: iron neodymium boride Nd_2_Fe_14_B.* Shoemaker, C. B., Shoemaker, D. P. & Fruchart, R. (1984). *Acta Cryst.* C**40**, 1665–1668. https://doi.org/10.1107/S0108270184009094.

A systematic study of a class of important industrial pigments: *Structures of eleven perylene-3,4:9,10-bis­(dicarboximide) pigments.* Hadicke, E. & Graser, F. (1986). *Acta Cryst.* C**42**, 189–195. https://doi.org/10.1107/S0108270186096828.

A study to classify structures of wurtzite materials, using neutron powder diffraction: *Upsilon-parameters for the wurtzite structure of ZnS and ZnO using powder neutron diffraction.* Kisi, E. H. & Elcombe, M. M. (1989). *Acta Cryst.* C**45**, 1867–1870. https://doi.org/10.1107/S0108270189004269.

An important paper on energetic materials relating solid-state structure to explosive properties: *1,1-Di­amino-2,2-di­nitro­ethyl­ene: a novel energetic material with infinite layers in two dimensions.* Bemm, U. & Ostmark, H. (1998). *Acta Cryst.* C**54**, 104–111. https://doi.org/10.1107/S0108270198007987.

An insightful polymorph study of a useful exemplar molecule for X-ray and electron diffraction: *Polymorphism in pentacene.* Mattheus, C. C., Dros, A. B., Baas, J., Meetsma, A., de Boer, J. L. & Palstra, T. T. M. (2001). *Acta Cryst.* C**57**, 939–941. https://doi.org/10.1107/S010827010100703X.

Computational studies of LiH_*x*_ superconductors for critical temperatures under pressure: *Superconductivity of lithium-doped hydrogen under high pressure.* Xie, Y., Li, Q., Oganov, A. R. & Wang, H. (2014). *Acta Cryst.* C**70**, 104–111. https://doi.org/10.1107/S2053229613028337.

A paper highlighting identification of pseudosymmetry in single-crystal X-ray diffraction: *Crystal structure and pseudosymmetry analysis of the triclinic prodrug cloxazolam (Z′ = 4).* Garraza, M. S., Gimenez, M. E., Vega, D. R. & Baggio, R. (2019). *Acta Cryst.* C**75**, 851–858. https://doi.org/10.1107/S2053229619008404.

A paper discussing issues relevant to the origins of coordination chemistry: *Serendipity: Werner’s argument that the ‘two-only forms’ (green and violet) of [CoCl_2_(en)_2_]^+^ salts demanded his octahedral model was correct. True?* Bernal, I. & Lalancette, R. A. (2020). *Acta Cryst.* C**76**, 298–301. https://doi.org/10.1107/S2053229620002272.

A gold-standard psilocybin study relating powder diffraction to density functional theory: *Psilocybin: crystal structure solutions enable phase analysis of prior art and recently patented examples.* Sherwood, A. M., Kargbo, R. B., Kaylo, K. W., Cozzi, N. V., Meisenheimer, P. & Kaduk, J. A. (2022). *Acta Cryst.* C**78**, 36–55. https://doi.org/10.1107/S2053229621013164.

### 2.5. *Acta Cryst. D: Structural Biology* and *Acta Cryst. F: Structural Biology Communications*

*Acta Cryst. D* continues to welcome articles covering any aspect of structural biology. It has always published papers on the structures of biological macromolecules, with emphasis on what they can tell us about biology backed up by other experimental methods. These have been accompanied by a mix of papers describing the theoretical underpinnings of structural biology, as well as the experimental methods and computer programs used, well cited by those in the structural biology field. Thus, some of the most cited papers describe large packages of computer software for solving, refining and interpreting the structures of protein and nucleic acids, as indicated below.

Most recent paper on the CCP4 software suite for macromolecular crystallography, with earlier versions in 1994 and 2011: *The CCP4 suite: integrative software for macromolecular crystallography.* Agirre, J., Atanasova, M., Bagdonas, H., Ballard, C. B., Baslé, A., Beilsten-Edmands, J., Borges, R. J., Brown, D. G., Burgos-Mármol, J. J., Berrisford, J. M., Bond, P. S., Caballero, I., Catapano, L., Chojnowski, G., Cook, A. G., Cowtan, K. D., Croll, T. I., Debreczeni, J. É., Devenish, N. E., Dodson, E. J., Drevon, T. R., Emsley, P., Evans, G., Evans, P. R., Fando, M., Foadi, J., Fuentes-Montero, L., Garman, E. F., Gerstel, M., Gildea, R. J., Hatti, K., Hekkelman, M. L., Heuser, P., Hoh, S. W., Hough, M. A., Jenkins, H. T., Jiménez, E., Joosten, R. P., Keegan, R. M., Keep, N., Krissinel, E. B., Kolenko, P., Kovalevskiy, O., Lamzin, V. S., Lawson, D. M., Lebedev, A. A., Leslie, A. G. W., Lohkamp, B., Long, F., Malÿ, M.,McCoy, A. J., McNicholas, S. J., Medina, A., Millán, C., Murray, J. W., Murshudov, G. N., Nicholls, R. A., Noble, M. E. M., Oeffner, R., Pannu, N. S., Parkhurst, J. M., Pearce, N., Pereira, J., Perrakis, A., Powell, H. R., Read, R. J., Rigden, D. J., Rochira, W., Sammito, M., Rodríguez, F. S., Sheldrick, G. M., Shelley, K. L., Simkovic, F., Simpkin, A. J., Skubak, P., Sobolev, E., Steiner, R. A., Stevenson, K., Tews, I., Thomas, J. M. H., Thorn, A., Valls, J. T., Uski, V., Usón, I., Vagin, A., Velankar, S., Vollmar, M., Walden, H., Waterman, D., Wilson, K. S., Winn, M. D., Winter, G., Wojdyr, M. & Yamashita, K. (2023). *Acta Cryst.* D**79**, 449–461. https://doi.org/10.1107/S2059798323003595.

Most recent paper on the widely used *Phenix* suite with earlier versions in 2002 and 2010: *Macromolecular structure determination using X-rays, neutrons and electrons: recent developments in Phenix.* Liebschner, D., Afonine, P. V., Baker, M. L., Bunkóczi, G., Chen, V. B., Croll, T. I., Hintze, B., Hung, L.-W., Jain, S., McCoy, A. J., Moriarty, N. W., Oeffner, R. D., Poon, B. K., Prisant, M. G., Read, R. J., Richardson, J. S., Richardson, D. C., Sammito, M. D., Sobolev, O. V., Stockwell, D. H., Terwilliger, T. C., Urzhumtsev, A. G., Videau, L. L., Williams C. J. & Adams, P. D. (2019). *Acta Cryst.* D**75**, 861–877. https://doi.org/10.1107/S2059798319011471.

*Coot* is a heavily used graphics program for mapping and manipulating atomic models: *Features and development of Coot.* Emsley, P., Lohkamp, B., Scott, W. G. & Cowtan, K. (2010). *Acta Cryst.* D**66**, 486–501. https://doi.org/10.1107/S0907444910007493.

*Refmac* is a powerful refinement program distributed with CCP4: *REFMAC5 for the refinement of macromolecular crystal structures.* Murshudov, G. N., Skubak, P., Lebedev, A. A., Pannu, N. S., Steiner, R. A., Nicholls, R. A., Winn, M. D., Long, F. & Vagin, A. A. (2011). *Acta Cryst.* D**67**, 355–367. https://doi.org/10.1107/S0907444911001314.

Macromolecular crystallography has benefited immensely by an early decision in the field to make structural data openly available through the Protein Data Bank (PDB). The existence of the PDB has allowed insights and new results to be gained through data mining.

One of the main PDB citation papers: *The Protein Data Bank.* Berman, H. M., Battistuz, T., Bhat, T. N., Bluhm, W. F., Bourne, P. E., Burkhardt, K., Feng, Z., Gilliland, G. L., Iype, L., Jain, S., Fagan, P., Marvin, J., Padilla, D., Ravichandran, V., Schneider, B., Thanki, N., Weissig, H., Westbrook, J. D. & Zardecki, C. (2002). *Acta Cryst.* D**58**, 899–907. https://doi.org/10.1107/S0907444902003451.

An example of data mining to show how metal can be identified bound to a protein: *Geometry of metal-ligand interactions in proteins.* Harding, M. M. (2001). *Acta Cryst.* D**57**, 401–411. https://doi.org/10.1107/S0907444900019168.

*Acta Cryst. D* has published many highly cited papers on structural biology methods other than computational as well as individual structural papers. Some examples follow.

Making the case for fine slicing in the rotation method for data collection when detector technology allowed: *The finer things in X-ray diffraction data collection.* Pflugrath, J. W. (1997). *Acta Cryst.* D**55**, 1718–1725. https://doi.org/10.1107/S090744499900935X.

A seminal protein production technique used in many subsequent studies: *A time- and cost-efficient system for high-level protein production in mammalian cells.* Aricescu, A. R., Lu, W. & Jones, E. Y. (2006). *Acta Cryst.* D**62**, 1243–1250. https://doi.org/10.1107/S0907444906029799.

Protein production techniques used in many subsequent studies: *A time- and cost-efficient system for high-level protein production in mammalian cells.* Aricescu, A. R., Lu, W. & Jones, E. Y. (2006). *Acta Cryst.* D**62**, 1243–1250. https://doi.org/10.1107/S0907444906029799.

A seminal paper on radiation damage issues: *Radiation damage in macromolecular crystallography: what is it and why should we care?* Garman, E. F. (2010). *Acta Cryst.* D**66**, 339–351. https://doi.org/10.1107/S0907444910008656.

A major paper on serum albumin providing insights on how it binds to many ligands: *Structures of bovine, equine and leporine serum albumin.* Bujacz, A. (2012). *Acta Cryst.* D**68**, 1278–1289. https://doi.org/10.1107/S0907444912027047.

Recent developments of importance published in *Acta Cryst. D* include the emergence of machine learning in structural biology discovery, such as covered in the following:

Feature article explaining how *AlphaFold2* works, by some of the authors of the open source *OpenFold* reimplementation: *Protein structure prediction by AlphaFold2: are attention and symmetries all you need?* Bouatta, N., Sorger, P. & AlQuraishi, M. (2021). *Acta Cryst.* D**77**, 982–991. https://doi.org/10.1107/S2059798321007531.

Meanwhile, *Acta Cryst. F* remains a rapid structural biology communications journal. The shorter papers published in *Acta Cryst. F* are intended to complement the scope of *Acta Cryst. D*. There are also longer papers, and several have been significant, attracting high numbers of citations. Some examples follow.

An overview of protein crystallization, including innovations and challenges: *Introduction to protein crystallization.* McPherson, A. & Gavira, J. A. (2014). *Acta Cryst.* F**70**, 2–20. https://doi.org/10.1107/S2053230X13033141.

A paper showing the importance of protein stability to optimize expression, purification, crystallization: *Protein stability: a crystallographer’s perspective.* Deller, M. C., Kong, L. & Rupp, B. (2016). *Acta Cryst.* F**72**, 72–95. https://doi.org/10.1107/S2053230X15024619.

A review of a crystallization method for membranes, soluble proteins *etc*., and applications for *in situ* serial crystallography: *A comprehensive review of the lipid cubic phase or in meso method for crystallizing membrane and soluble proteins and complexes.* Caffrey, M. (2015). *Acta Cryst.* F**71**, 3–18. https://doi.org/10.1107/S2053230X14026843.

A paper surveying the scale and growth of biological database (functionally) uncharacterized families (DUFs) and prospects for discovering their functions: *DUFs: families in search of function.* Bateman, A., Coggill, P. & Finn, R. D. (2010). *Acta Cryst.* F**66**, 1148–1152. https://doi.org/10.1107/S1744309110001685.

A paper presenting the crystal structure of the full-length Zika virus NS5 protein at 3.05 Å resolution, which may facilitate the structure-based design of antiviral agents against ZIKV: *Crystal structure of full-length Zika virus NS5 protein reveals a conformation similar to Japanese encephalitis virus NS5.* Upadhyay, A. K., Cyr, M., Longenecker, K., Tripathi, R., Sun, C. H. & Kempf, D. J. (2017). *Acta Cryst.* F**73**, 116–122. https://doi.org/10.1107/S2053230X17001601.

A protocol for recombinant production, purification and crystallization of a protein together with a high-resolution crystal structure key to understanding its biological role: *The structure of a tautomerase superfamily member linked to the type VI secretion system of Acinetobacter baumannii.* Pankov, G., Mol Avelar, G., Buchanan, G., Coulthurst, S. J. & Hunter, W. N. (2023). *Acta Cryst.* F**79**, 8–16. https://doi.org/10.1107/S2053230X22011414.

*Acta Cryst. F* has also published papers on COVID-19 and has carried a recent Editorial on new machine-learning methods, such as *AlphaFold*, while also outlining the continuing need for experimental structural biology:

*Room-temperature neutron and X-ray data collection of 3CL M-pro from SARS-CoV-2.* Kneller, D. W., Phillips, G., Kovalevsky, A. & Coates, L. (2020). *Acta Cryst.* F**76**, 483–487. https://doi.org/10.1107/S2053230X20011814.

*AlphaFold and the future of structural biology.* Read, R. J., Baker, E. N., Bond, C. S., Garman, E. F. & van Raaij, M. J. (2023). *Acta Cryst.* F**79**, 166–168. https://doi.org/10.1107/S2053230X23004934.

### 2.6. *Acta Cryst. E: Crystallographic Communications* and *IUCrData*

*Acta Cryst. E* has become the IUCr’s open-access structural communications journal and has regained a full citation indexing in the listings published in 2023, while *IUCrData* is IUCr’s peer-reviewed open-access data journal. *Acta Cryst. E* remains one of IUCr’s most popular journals measured by numbers of submissions. It also has increasing international outreach for developing countries in crystallography and structural science, with significant potential for growth in teaching and education at the international level. In this connection, two recent papers in *Acta Cryst. E* have been particularly highly cited:

*Utilizing Hirshfeld surface calculations, non-covalent inter­action (NCI) plots and the calculation of inter­action energies in the analysis of molecular packing.* Tan, S. L., Jotani, M. M. & Tiekink, E. R. (2019). *Acta Cryst.* E**75**, 308–318. https://doi.org/10.1107/S2056989019001129.

*CheckCIF validation alerts: what they mean and how to respond.* Spek, A. L. (2020). *Acta Cryst.* E**76**, 1–11. https://doi.org/10.1107/S2056989019016244.

While *IUCrData* is not an indexed journal in the usual sense, it plays an important role in data publication. Given the greater demands for open data associated with scientific research publication (see below), *IUCrData* has the potential to develop in several ways over the coming years. To address one such area, a new section has recently been introduced for *IUCrData*, namely *Raw Data Letters*, as explained in a recent editorial paper:

*IUCrData launches Raw Data Letters.* Kroon-Batenburg, L. M. J., Helliwell, J. R. & and J. R. Hester, J. R. (2022). *IUCrData*, **7**, x220821. https://doi.org/10.1107/S2414314622008215.

### 2.7. Journal of Applied Crystallography

The *Journal of Applied Crystallography* publishes papers on X-ray, neutron or electron-based applied structural science across condensed matter research, materials science and life sciences, including instrumentation, facilities, and *validated* computer software and programs. It has published significant and highly cited papers every year since its inception, of which a brief selection follows.

One of the seminal papers on the Rietveld method, here for neutron powder diffraction: *A profile refinement method for nuclear and magnetic structures.* Rietveld, H. M. (1969). *J. Appl. Cryst*, **2**, 65–71. https://dx.doi.org/10.1107/S0021889869006558.

A paper reporting the first cryostat for widespread general use at X-ray diffraction facilities: *A nitro­gen-gas-stream cryostat for general X-ray diffraction studies.* Cosier, J. & Glazer, A. M. (1986). *J. Appl. Cryst*, **19**, 105–107. https://doi.org/10.1107/S0021889886089835.

A semi-invariants representation (*SIR*) software package developed for solving crystal structures by direct methods: *SIR88 - a direct-methods program for the automatic solution of crystal structures.* Burla, M. C., Camalli, M., Cascarano, G., Giacovazzo, C., Polidori, G., Spagna R. & Viterbo, D. (1989). *J. Appl. Cryst.* **22**, 389–393. https://doi.org/10.1107/S0021889889004103. An update: *SIR97: a new tool for crystal structure determination and refinement.* Altomare, A., Burla, M. C., Camalli, M., Cascarano, G. L., Giacovazzo, C., Guagliardi, A., Moliterni, A. G. G., Polidori, G. & Spagna, R. (1999). *J. Appl. Cryst.* **32**, 115–119. https://doi.org/10.1107/S0021889898007717. Also, later updates: *SIR2004*, *SIR2011*, *SIR2014* *etc*.

The seminal paper on grazing-incidence small-angle X-ray scattering (GISAXS): *Grazing-incidence small-angle X-ray scattering: new tool for studying thin film growth.* Levine, J. R., Cohen, J. B., Chung Y. W. & Georgopoulos, P. (1989). *J. Appl. Cryst.* **22**, 528–532. https://doi.org/10.1107/S002188988900717X.

A protein visualization program for schematic ribbon diagrams of α-helices and β-sheets: *MOLSCRIPT: a program to produce both detailed and schematic plots of protein structures.* Kraulis, P. J. (1991). *J. Appl. Cryst.* **24**, 946–950. https://doi.org/10.1107/S0021889891004399.

A program package for single-crystal diffraction data, structure refinement and publication: *WinGX suite for small-molecule single-crystal crystallography.* Farrugia, L. J. (1999). *J. Appl. Cryst.* **32**, 837–838. https://doi.org/10.1107/S0021889899006020. Also: *WinGX and ORTEP for Windows: an update.* Farrugia, L. J. (2012). *J. Appl. Cryst.* **45**, 849–854. https://doi.org/10.1107/S0021889812029111.

Program validation tools for IUCr Crystallographic Information Files (CIFs): *Single-crystal structure validation with the program PLATON.* Spek, A. L. (2003). *J. Appl. Cryst.* **36**, 7–13. https://doi.org/10.1107/S0021889802022112.

The most-widely used software for processing of serial femtosecond crystallography data: *CrystFEL: a software suite for snapshot serial crystallography.* White, T. A., Kirian, R. A., Martin, A. V., Aquila, A., Nass, K., Barty, A. & Chapman, H. N. (2012). *J. Appl. Cryst.* **45**, 335–341. https://doi.org/10.1107/S0021889812002312. Also, 2016 update.

One of the most downloaded *Teaching and Education* papers: *Crystallographic education in the 21st century.* Gražulis, S., Sarjeant, A. A., Moeck, P., Stone-Sundberg, J., Snyder, T. J., Kaminsky, W., Oliver, A. G., Stern, C. L., Dawe, L. N., Rychkov, D. A., Losev, E. A., Boldyreva, E. V., Tanski, J. M., Bernstein, J., Rabeh, W. M. & Kantardjieff, K. A. (2015). *J. Appl. Cryst.* **48**,1964–1975. https://dx.doi.org/10.1107/S1600576715016830.

A paper demonstrating interference from separate crystals in a thermal neutron interferometer: *Neutron interference from a split-crystal interferometer*. Lemmel, H., Jentschel, M., Abele, H., Lafont, F., Guerard, B., Sasso, C. P., Mana G. & Massa, E. (2022). *J. Appl. Cryst.* **55**, 870–875. https://doi.org/10.1107/S1600576722006082. The instrument S18 at the Institut Laue Langevin, Grenoble, France, reported here, was used by Anton Zeilinger for part of his 2022 Nobel Physics Prize work – see https://www.ill.eu/news-press-events/news/general-news/nobel-prize-in-physics-2022-for-anton-zeilinger-the-ill-congratulates.

A first X-ray imaging study of current crowding in fully packaged integrated circuit chips: *X-ray diffraction imaging of fully packaged n–p–n transistors under accelerated ageing conditions.* Tanner, B. K., Danilewsky, A. & McNally, P. J. (2022). *J. Appl. Cryst.* **55**, 1139–1146. https://doi.org/10.1107/S1600576722007142.

Recent application of machine learning to serial crystallography data handling: *Data reduction for X-ray serial crystallography using machine learning.* Rahmani, V., Nawaz, S., Pennicard, D., Setty, S. P. R. & Graafsma, H. (2023). *J. Appl. Cryst.* **56**, 200–213. https://doi.org/10.1107/S1600576722011748.

From its inception, the *Journal of Applied Crystallography* has played a critical role in building up the small-angle (X-ray and neutron) scattering (SAS) research community and the formation of the IUCr Commission on SAS, with Special Issues associated with the Triennial SAS Conferences and many significant highly cited articles, of which the two listed below are representative:

*Uniqueness of ab initio shape determination in small-angle scattering.* Volkov, V. V. & Svergun, D. I. (2003). *J. Appl. Cryst.* **36**, 860–864. https://doi.org/10.1107/S0021889803000268.

*Approximations leading to a unified exponential power-law approach to small-angle scattering.* Beaucage, G. (1995). *J. Appl. Cryst.* **28**, 717–728. https://doi.org/10.1107/S0021889895005292.

### 2.8. Journal of Synchrotron Radiation

The *Journal of Synchrotron Radiation* provides comprehensive coverage of the entire field of synchrotron radiation and X-ray free-electron laser (XFEL) research. A selection of its most significant and highly cited papers from over the years follows.

Design and performance of a dynamical sagittal-focusing monochromator for hard X-rays: *X-ray optics of a dynamical sagittal-focusing monochromator on the GILDA beamline at the ESRF.* Pascarelli, S., Boscherini, F., D’Acapito, F., Hrdy, J., Meneghini, C. & Mobilio, S. (1996). *J. Synchrotron Rad*. **3**, 147–155. https://doi.org/10.1107/S0909049596004992.

Design of a synchrotron-based single-crystal X-ray diffraction facility for materials science: *A new high-flux chemical and materials crystallography station at the SRS Daresbury. 1. Design, construction and test results.* Cernik, R. J., Clegg, W., Catlow, C. R. A., Bushnell-Wye, G., Flaherty, J. V., Greaves, G. N., Burrows, I., Taylor, D. J., Teat, S. J. & Hamichi, M. (1997). *J. Synchrotron Rad.* **4**, 279–286. https://doi.org/10.1107/S090904959701008X.

Versatile X-ray absorption spectroscopy software adaptable for synchrotron experiments: *WinXAS: a program for X-ray absorption spectroscopy data analysis under MS-Windows.* Ressler, T. (1998). *J. Synchrotron Rad.* **5**, 118–122. https://doi.org/10.1107/S0909049597019298.

Manufacture and properties of novel compound refractive lenses for hard X-ray applications: *Imaging by parabolic refractive lenses in the hard X-ray range.* Lengeler, B., Schroer, C., Tümmler, J., Benner, B., Richwin, M., Snigirev, A., Snigireva, I. & Drakopoulos, M. (1999). *J. Synchrotron Rad.* **6**, 1153–1167. https://doi.org/10.1107/S0909049599009747.

A major application program to calculate various characteristics of synchrotron radiation: *SPECTRA: a synchrotron radiation calculation code.* Tanaka, T. & Kitamura, H. (2001). *J. Synchrotron Rad.* **8**, 1221–1228. https://doi.org/10.1107/S090904950101425X.

A study showing how state-of-the-art experiments in the soft X-ray energy range remain important: *Interferometer-controlled scanning transmission X-ray microscopes at the Advanced Light Source.* Kilcoyne, A. L. D., Tyliszczak, T., Steele, W. F., Fakra, S., Hitchcock, P., Franck, K., Anderson, E., Harteneck, B., Rightor, *e.g.*, Mitchell, G. E., Hitchcock, A. P., Yang, L., Warwick, T. & Ade, H. (2003). *J. Synchrotron Rad.* **10**, 125–136. https://doi.org/10.1107/S0909049502017739.

Widely used data analysis codes revolutionizing the X-ray spectroscopy field at synchrotrons: *ATHENA, ARTEMIS, HEPHAESTUS: data analysis for X-ray absorption spectroscopy using IFEFFIT.* Ravel, B. & Newville, M. (2005). *J. Synchrotron Rad.* **12**, 537–541. https://doi.org/10.1107/S0909049505012719.

A scientific account of a revolutionary new detector development in the synchrotron field: *The Pilatus 1M detector.* Broennimann, C. H., Eikenberry, E. F., Henrich, B., Horisberger, R., Huelsen, G., Pohl, E., Schmitt, B., Schulze-Briese, C., Suzuki, M., Tomizaki, T., Toyokawa, H. & Wagner, A. (2006). *J. Synchrotron Rad.* **13**, 120–130. https://doi.org/10.1107/S0909049505038665.

New detector technology for X-ray free-electron laser facilities: *The adaptive gain integrating pixel detector at the European XFEL.* Allahgholi, A., Becker, J., Delfs, A., Dinapoli, R., Goettlicher, P., Greiffenberg, D., Henrich, B., Hirsemann, H., Kuhn, M., Klanner, R., Klyuev, A., Krueger, H., Lange, S., Laurus, T., Marras, A., Mezza, D., Mozzanica, A., Niemann, M., Poehlsen, J., Schwandt, J., Sheviakov, I., Shi, X., Smoljanin, S., Steffen, L., Sztuk-Dambietz, J., Trunk, U., Xia, Q., Zeribi, M., Zhang, J., Zimmer, M., Schmitt, B. & Graafsma, H. (2019). *J. Synchrotron Rad.* **26**, 74–82. https://doi.org/10.1107/S1600577518016077.

Development of time-resolved diffraction/imaging at the first fourth-generation hard X-ray source: *ID15A at the ESRF – a beamline for high-speed operando X-ray diffraction, diffraction tomography and total scattering.* Vaughan, G. B. M., Baker, R., Barret, R., Bonnefoy, J., Buslaps, T., Checchia, S., Duran, D., Fihman, F., Got, P., Kieffer, J., Kimber, S. A. J., Martel, K., Morawe, C., Mottin, D., Papillon, E., Petitdemange, S., Vamvakeros, A., Vieux, J.-P. & Di Michiel, M. (2020). *J. Synchrotron Rad.* **27**, 515–528. https://doi.org/10.1107/S1600577519016813.

The most cited paper in the *Journal of Synchrotron Radiation* in the last year: *I21: an advanced high-resolution resonant inelastic X-ray scattering beamline at Diamond Light Source.* Zhou, K.-J., Walters, A., Garcia-Fernandez, M., Rice, T., Hand, M., Nag, A., Li, J., Agrestini, S., Garland, P., Wang, H., Alcock, S., Nistea, I., Nutter, B., Rubies, N., Knap, G., Gaughran, M., Yuan, F., Chang, P., Emmins, J. & Howell, G. (2022). *J. Synchrotron Rad.* **29**, 563–580. https://doi.org/10.1107/S1600577522000601.

The *Journal of Synchrotron Radiation* is now fully open access and continues to publish significant papers of interest as new diffraction-limited storage ring synchrotrons and X-ray free-electron laser facilities come online. At the same time, the *Journal of Synchrotron Radiation* also covers the emergence of facilities in the developing world. Typical recent papers are listed below.

*X-ray in-line holography and holotomography at the NanoMAX beamline.* Kalbfleisch, S., Zhang, Y. H., Kahnt, M., Buakor, K., Langer, M., Dreier, T., Dierks, H., Stjarneblad, P., Larsson, E., Gordeyeva, K., Chayanun, L., Soderberg, D., Wallentin, J., Bech, M. & Villanueva-Perez, P. (2022). *J. Synchrotron Rad.* **29**, 224–229. https://doi.org/10.1107/S1600577521012200.

*3D printed devices and infrastructure for liquid sample delivery at the European XFEL*. Vakili, M., Bielecki, J., Knoska, J., Otte, F., Han, H. J., Kloos, M., Schubert, R., Delmas, E., Mills, G., de Wijn, R., Letrun, R., Dold, S., Bean, R., Round, A., Kim, Y., Lima, F. A., Dorner, K., Valerio, J., Heymann, M. & Mancuso, A. P. (2022). *J. Synchrotron Rad.* **29**, 331–346. https://doi.org/10.1107/S1600577521013370.

*Operational status of the X-ray powder diffraction beamline at the SESAME synchrotron.* Abdellatief, M., Al Najdawi, M., Momani, Y., Aljamal, B., Abbadi, A., Harfouche, M. & Paolucci, G. (2022). *J. Synchrotron Rad.* **29**, 532–539. https://doi.org/10.1107/S1600577521012820.

## 3. Challenges and opportunities for the future

For 75 years the IUCr journals have worked in tandem with the IUCr, its Commissions, its Affiliates and its National Committees, to establish, deepen, broaden and expand a large international record of peer-reviewed crystallography-based structural science research. Much of this endeavour is demonstrated in the titles and authors of well cited papers selected for the above highlights. As a number of these papers also show over the years, IUCr journals have played major roles in facilitating curated databases, establishing standards and validation tools for CIF files and other structure reports, and presenting methodologies, facilities and software capabilities for structural data acquisition, reduction, analysis and interpretation. Additionally, important papers have appeared dedicated explicitly to training and educating new generations of crystallographers and researchers in structural science.

Despite these achievements, the IUCr journals, like all scientific research journals, must navigate the rapidly changing landscape for scientific publication in general. The almost complete transformation from paper hardcopy publication to digital publication (and digital review processes), together with the increased prominence of competitive, commercially funded, scientific journals (and sometimes a correspondingly reduced role of scientific societies), are obvious examples from recent decades (Strickland & McMahon, 2008 ▸; Strickland & Allen, 2021 ▸). However, the ongoing transformations to provide open-access publication (Young & Brandes, 2020 ▸; cOAlition S, 2020 ▸; Kvashnina *et al.*, 2021 ▸), findable, accessible interoperable re-usable (FAIR), open data (Wilkinson *et al.*, 2016 ▸; Helliwell *et al.*, 2019 ▸), and open science in general (International Science Council, 2021 ▸; UNESCO, 2021 ▸), are likely to have a far more profound impact on sustainable publishing models over the next few years. While, currently, six hybrid IUCr journals allow authors a ‘gold’ open-access option (see below) but also support the ‘green’ open-access model (reproduction of the final accepted form of a paper in a public repository), four IUCr journals are already fully ‘gold’ open access with all published journal papers freely available following payment of an article publishing charge (APC). This is paid either by the corresponding author’s institution or through an institutional publishing ‘read and publish’ agreement. Although emphasizing that any decisions have yet to be made, it is not unreasonable to speculate that more of the journals may flip to fully (gold) open access within the next decade.

Other current challenges include the effects of the war in Ukraine, which has inevitably reduced submissions from that part of the world. There have also been some delayed effects of the COVID-19 pandemic, where many researchers, denied access to their experimental facilities, initially used the opportunity to prepare and submit papers based on previous measurements, as well as publish research relevant to the pandemic itself (including some among those listed above). However, the various neutron and X-ray facility shutdowns, some starting or continuing after the pandemic for major upgrades, do appear to have slowed overall publication submission rates at the present time, as reported at several facility user meetings internationally. This situation will recover and likely be followed by many exciting submissions to the IUCr journals in coming years. Other long-term challenges include bringing new generations of researchers and students into crystallography and structural science in general, and introducing new initiatives that will ensure the IUCr journals remain competitive, successful and sustainable in the long term in an emerging era of open science. Several recent and ongoing developments are designed to address these issues.

Active commissioning both of high-quality individual articles and Special Issues has become an increasingly significant activity for all involved with the journals. Starting in 2019, Commissioning Editors have been appointed to serve across multiple IUCr journals in three main areas of interest: *Biological Sciences* (mainly with *Acta Cryst. D* and *F*), *Chemical Sciences* (mainly with *Acta Cryst. B*, *C* and *E*), and *Materials, Methods and Instrumentation* (mainly with *Acta Cryst. A*, *Journal of Applied Crystallography* and *Journal of Synchrotron Radiation*). All three Commissioning Editors interact as appropriate with *IUCrJ* and *IUCrData* editors. The Commissioning Editors work with the Journal Main and Managing Editors to commission new Special Issues on topics of current interest, as well as individual articles, *e.g.* Feature and Lead Articles, by expert leaders in new fields of interest to the IUCr research community. A major advantage of having Commissioning Editors work across several journals, coupled with the online digital nature of the journals, is that Special Issues can themselves be shared across journals when it makes sense to do so. Papers undergo the full review process for one of the journals, are published in a regular issue if accepted with full citation information for their original publication, then all Special Issue papers are *also* collected together into the virtual Special Issue with a Foreword written by the Special Issue Guest Editors, introducing the Special Issue and providing links to the papers. A retrospective Special Collection of papers recently published across multiple journals can also be assembled with an appropriate Foreword to form a virtual Special Collection in a similar way. While some Special Issue series remain associated with conference series of interest to crystallographers, these are no longer Conference Proceedings in the traditional sense, due to conference papers (usually) having lower citation rates than regular papers. Instead, Special Issue papers go through the full review process for the relevant journal and, for many successful Special Issues, the published articles are more highly cited than regular papers published in the journal.

Commissioning activities and Special Issues keep the journals active in current and new areas of interest and introduce new authors and potential editors to the IUCr journals over time. They also allow new subject areas to be explored for incorporation into the permanent scope of each journal, maintaining relevance and competitiveness with non-IUCr journals in the field. The new subject area sections added to *IUCrJ* in *Electron Microscopy*, to *Acta Cryst. B* in *Crystal Growth* and to *IUCrData* in *Raw Data Letters* are recent examples. Another area of note is the increased effort across several of the journals to support *Teaching and Education* initiatives, especially in developing regions of the world, in tandem with the IUCr’s more general outreach activities.

Virtual Editorial Board meetings (Main Editors, Managing Editor, Co-editors, Editor-in-Chief) have recently been instituted for each journal. Commissioning activities, new subject areas and many other aspects of each journal, from its editorial and review system, mentoring of new Co-editors, to journal publicity, can be discussed informally at such meetings. However, the main forum for guiding development of the IUCr journals, overall, remains the Journals Management Board (JMB), instituted by my predecessor, comprising Main Editors, Managing Editors, Editorial Office staff, the Editor-in-Chief and the IUCr President. The JMB meets in person, at least once per year, usually in Chester, UK. All issues pertaining to journal performance, editorial processes and journals development are discussed at the JMB, and reported to both the IUCr Finance Committee and the IUCr Executive Committee, the latter approving all executive decisions and confirming editorial appointments. In regard to editorial appointments, as well as providing the appropriate subject coverage for each journal, there is a strong and ongoing effort in recent years to appoint Editors and Co-editors who provide diversity in gender and geography to reflect the active international structural science community.

In closing, it is important to state that the success of the IUCr journals over the last 75 years has depended critically on a large number of dedicated researchers in crystallography and structural science. They continue to provide many volunteer hours to the journals as Co-editors, Main Editors, Commissioning Editors, reviewers and sometimes consultants. Many are frequent prominent authors, themselves, of high-quality papers published in the journals, and some contribute to the IUCr in other major ways. This enormous effort is complemented by the dedicated work of the staff in the Chester, UK, Editorial Office. Together, this combination provides a publication experience to authors and readers alike, that in many ways is unique. Whatever changes are in store for the IUCr journals in the coming years, it is this combination of dedicated talent that will be at the heart of the IUCr journals success over the next 75 years.

## Footnotes

1 Certain commercial instruments, materials, software or processes are identified in this paper to adequately specify the experimental procedure. Such identification does not imply recommendation or endorsement by the National Institute of Standards and Technology, nor does it imply that the instruments, materials or processes identified are necessarily the best available for the purpose.
